# The Anatomy and Philosophy of Expression, as Connected with the Fine Arts

**Published:** 1844-10

**Authors:** 


### ART. XIII.

*The Anatomy and Philosophy of Expression, as connected with the Fine Arts.* By SIR CHARLES BELL, K.H. Third Edition, enlarged. —*London*, 1844. Imp. 8vo, Plates, pp. 266.

ANATOMY and Physiology have important relations to the arts of design, less extensive perhaps, but certainly not less immediate and essential, than those which they bear to the arts of medicine and surgery. These relations have been little appreciated by our academies and schools of design. An attentive examination of the beautiful and perfect forms of the antique marbles, or of the works of such men as Michael Angelo Buonarotti, will indeed show that the importance of the study has been sufficiently appreciated in all ages, by men of genius; and that the obstacles which they must have encountered in the acquisition of such a knowledge of anatomy as their works display, were surmounted by efforts of patient study and minute observation, such as genius alone could make. But a reference to the means of anatomical study afforded to our artists both at home and abroad, will satisfy any one, on reflection, that its true value is not understood, and that such a knowledge as those means can supply will never subserve the purpose of the artist in the higher departments of painting and sculpture. "The academies of Europe," says the author of the work before us, "instituted for the improvement of painting, stop short of the science of anatomy, which is so well suited to enlarge the mind, and to train the eye for observing the forms of nature; or if they enforce the study at all, it is only in its more obvious application, that of assisting the drawing of the human figure." The study of "the round," of the smooth, and undulating surfaces of the antique, from plaster casts; or the drawing of the external form, from the spiritless and jaded academy figure; or a few popular lectures on the superficial muscles of the human body, can never confer that knowledge which the artist requires. An accurate knowledge of the skeleton, a familiar acquaintance with the form of every bone and articulation, and of the mechanism of the organs of locomotion, passive as well as active, is essentially necessary to enable him to design with freedom and accuracy the human form in the various actions and attitudes of which it is susceptible. He must not only know the form and situation of muscles and knobs of bone, but the mechanism of each movement, and the associated muscular actions with which each is necessarily accompanied. Nor is this all; he must study the muscular acts which are associated with mental emotions, not those alone which are immediately expressive of them, or called into voluntary exercise by their influence, but those which are excited into instinctive and involuntary action through the influence of the mind on the organs of circulation and respiration.

The application of anatomy to the arts of design extends still further; it includes a knowledge of the peculiarities which distinguish the countenance or body in every situation of interest to the painter and sculptor. "A knowledge of the peculiarities of country, of infancy, youth or age; of sickness or robust health; or of the contrasts between manly and muscular strength and feminine delicacy; or of the appearances which pain or death present, belongs to its province, as much as the study of the muscles of the face when affected in emotion."

These propositions are beautifully and forcibly illustrated in the concluding essays of the elegant and pleasing work under review.

"When I have seen," says its accomplished author, "one unacquainted with the internal structure, drawing from the naked figure or from a statue, I have remarked the difficulty he experienced in showing the course of a swelling muscle, or the slight depressions and conversities about a joint; and this difficulty might be traced to his ignorance of the relations and actions of the muscles. The same perplexity he often feels in drawing the knobbed ends of bones, or the insertions of the tendons at the articulations; for these parts being covered over by the integuments, and cushions of fat of variable thickness, and sheathed in membranes are but faintly marked on the surface. The delicate and less definite indications of the anatomy, though easily traced by one acquainted with the structure of the limb, appear to the uninformed only unmeaning variations in the outline; he has no means of judging of their importance; and he is subject to continual mistakes in attempting to imitate them. .... Drawing what he does not understand, he falls into lameness, or deviates into caricature. ...... Even for attaining a correct knowledge of the body and limbs, the academy figure is far from being an infallible guide. The display of muscular action in the human figure is but momentary, and cannot be retained and fixed for the imitation of the artist. The effect produced upon the surface of the body and limbs—the swelling and receding of the fleshy parts, and that starting out of the sinews or tendons which accompany exertion or change of posture cannot be observed with sufficient accuracy, unless the artist is able to class the muscles engaged in the action; and he requires some other guide to enable him to recollect these varying forms, than that which is afforded by a transitory view of them." (pp. 218-9.)

It may be doubted, however, whether the study of anatomy and physiology will furnish the artist with rules by which he may attain the highest objects of design, but it will educate his eye for the intelligent observation of those minute details, upon which the expression and truth of his delineations must depend.

The concluding essay of this work, which formed the introductory one of the former editions, is full of important hints, showing the utility of the study of anatomy to the painter and sculptor, and the extent to which the study may assist them in their designs. In the essay which precedes this one, a critique on the noble works of Michael Angelo, the result of the author's visit to the continent in 1840, finely illustrates the utility of anatomy in giving freedom, truth and power, to the designs of the artist.

The aim of the author of these essays was, however, of a higher kind, than that of merely commending the study of anatomy to the artist, and of illustrating its advantages. His design was "to direct attention to the characteristic forms of man and brutes, by an inquiry into the natural functions, with a view to comprehend the *rationale* of those changes in the countenance and figure which are indicative of passion." His work comprises an examination into the sources of beauty in the antique statues, and the theories of beauty, natural and ideal, in the human form. So far his work is a contribution to philosophy and the fine arts. But his design extended beyond this. He endeavoured to discover the laws which regulate the expression of the passions in the muscular movements of the countenance and whole frame. It was in the prosecution of these inquiries that Sir Charles Bell was led to the discovery of the motor and sensific tracts of the nervous system, and to his ingenious and beautiful theory of the respiratory system of nerves. To this work then, begun, as we are informed in the preface, and as he had fondly stated in a delineation designed for his brother, the late Professor of Scots law, " when we studied together before the serious pursuits of life began," we owe the highly important discoveries of Sir Charles Bell on the nervous system, and all to which they have subsequently led.

These essays formed not only the earliest, but through life the favourite, and, as it unfortunately chanced, the latest occupation of the leisure hours of their amiable and accomplished author. Originally published in 1806, long after they were begun, and republished again in 1824, they were almost entirely recomposed after the author's return from abroad. He visited the continent in 1840, for the purpose, as we are informed, of " verifying in Italy the principles of criticism in art by the study of the works of the great masters in painting and sculpture." It was while on the eve of finally revising this work for the press, during the relaxation from his professional duties afforded by the summer recess, that his life was terminated by a sudden illness.

Although the work is now issued to the public under the disadvantages which must always attend a posthumous publication, it has been edited with much care and judgment by his friend and relative, Mr. Shaw, of the Middlesex Hospital. That part of the work which was not re-written—namely, the essay on the nervous system—has been supplied by an appendix by Mr. Shaw, containing a history of the discoveries and theories of Sir Charles in relation to this subject. Those remarks contained in the author's private journal of his continental tour, not already embodied by himself in the work, but apparently designed for its illustration, have been appended in the form of notes.

Several new illustrative sketches, of great beauty and spirit, from designs by the author, have been introduced into this edition. Of these we would notice in particular the child's head in p. 17, as extremely beautiful. All the illustrations of the former editions which were really good have been retained; while those which were at all objectionable have been withdrawn.

In the introduction the author sketches a comparison between ancient and modern art. Setting aside the influence of climate, and of original difference in genius, he seeks for an explanation of their comparative merits in the influences of government, religion, and external agencies. The free institutions of ancient Greece were favorable to the development of genius; the athletic exercises and the national games of the country afforded the means of study to their artists, and a standard of taste and judgment to the public, while their mythology exercised a peculiar influence over the works of art. In the representation of their deities they were led to exaggerate all that was expressive of the higher and nobler qualities of man, and to subdue all that was expressive of sensual or animal passions, or even of human emotion, as far as it might be associated with human weakness. Hence, according to the theory of the author, the idea of *divinity* fancifully attributed to the beautiful forms of the antique.

The ancient Italian masters studied under different influences. The objects of representation were with them strictly human. With the models before them afforded by the antique marbles, they sought, indeed, in their representations of the Saviour, "to infuse *divinity* into the *human* beauty of that countenance, which, though not without feeling, was superior to passion, and in which benevolence was to be represented unclouded by human infirmity." But still it was the representation of *humanity*, not uninfluenced by human sympathies, infirmities, and sufferings. And in the wide range afforded to them by Scripture subjects, they had full scope for the representation of every variety of human character and expression. They studied, too, under the influence of a ceremonial which presented to their eyes rich and pleasing combinations of light and shade and colour, a grand and imposing architecture, picturesque groups in gorgeous robes, and rites hallowed by sacred associations, solemn music, and the smoke of incense.

Our artists study under less favorable auspices. In these northern climes the naked form is never seen, unless in the pale and shivering academy figure: fashion and civilization have destroyed all that can be considered picturesque or graceful in the colours or forms of our dress; while the religion of the Reformation and the influence of philosophy have removed the aids of external and sensible objects, and left the worshippers to the influence of internal contemplation founded on reason, and the exercise of faith in things unseen.

The first essay is devoted to an examination of the permanent form and relative proportions of the cranium and face; and leads the author, in his preliminary remarks and subsequent deductions, to an inquiry into the sources of beauty in the human countenance. In reviewing the theories of beauty, he rejects altogether the notion that there can exist in the imagination an ideal beauty, superior to or different from anything which may be seen in nature. He regards as easily fanciful the idea that beauty in art depends on avoiding what is human and aiming at the representation of the divine. He observes, with truth, that the idea of representing divinity is palpably absurd; and that we know nothing of form but from the contemplation of *man*. The representation of divinity was accomplished by the ancient sculptors, not by avoiding what was human, but by avoiding what was mean or weak in the human character, and exaggerating the features which were associated with the higher qualities and nobler emotions of the mind. This leads the author to the prevalent opinion, "that beauty of countenance consists in the capacity of expression, and in the harmony of the features consenting to that expression." An opinion more concisely expressed in the generalization so beautifully illustrated in Alison's Essays on the Nature and Principles of Taste, that beauty arises from association: those features or those expressions which are associated by us with the possession of pleasing or ennobling qualities, are beautiful or noble. With how much truth and beauty is the subject illustrated by the following paragraph, so characteristic of its author:

"A countenance may be distinguished by being expressive of thought; that is, it may indicate the possession of the intellectual powers. It is manly, it is human; and yet not a motion is seen to show what feeling or sentiment prevails. On the other hand, there may be a movement of the features, and the quality of thought—affection, love, joy, sorrow, gratitude, or sympathy with suffering—is immediately expressed. A countenance which, in ordinary conditions, has nothing remarkable, may become beautiful in expression. It is expression which raises affection, which dwells pleasantly or painfully on the memory. When we look forward to the meeting with those we love, it is the illuminated face we hurry to meet; and none who have lost a friend but must acknowledge that it is the evanescent expression, more than the permanent form, which is painfully dear to them." (Alison, p. 20.)

The theory of Sir Charles, somewhat differently expressed, does not differ essentially from the one just referred to. It is constructed with reference to the mode in which he has examined the subject. "Beauty," he says, "in the human form has relation to the characteristic organs of man." This principle is fully illustrated by an examination of the relative development of the face and cranium in man and in the lower animals, by a reference to the antique, and to the sources of expression characteristic of *human* passion, as contrasted with those expressive of animal propensities.

With regard to the relative development of the cranium and face, Sir Charles shows that the facial angle of Camper is not only deficient, as is generally acknowledged, as a means of estimating this comparative development, but that it is defective as a rule to the artist for enabling him to give dignity or beauty to the countenance, by merely throwing forward the forehead.

"Camper's position is this: that as by the diminution of the cranium, and the further inclination of the facial line the head is depressed in character to that of the Negro; so, by raising and throwing the skull upwards and forwards, until the facial line reaches the perpendicular, the great object is attained of resemblance to the antique head. But his own figures contradict his conclusion; for although he has thrown the head forward in them, even beyond the perpendicular of the facial line, yet, as he has preserved the features of common nature, we refuse to acknowledge their similarity to the beautiful forms of the antique marbles. It is true that by advancing the forehead it is raised; the face is shortened, and the eye brought to the centre of the head. But, with all this, there is much wanting —that which measurement or mere line will not show us." (pp. 28-9.)

Illustrative sketches are given, affording abundant evidence of the justice of these remarks. The sketches from Camper completely stultify his own theory. The face in which the facial angle has been increased to more than 90°, is no more to be compared with the antique than "Hyperion to a satyr!" As shown in another sketch, deformity of the bones, from rickets, may increase the angle, even beyond the right angle, as is the case in the Jupiter Tonans, without producing any other idea than that of deformity.

From a comparison of the area of the cranium with that of the face, as seen in a vertical section from before backwards, the mode of comparison recommended by Cuvier, the general law deduced by anatomists, is that the cranium and face, in the different varieties of man, and in the mammalia, are developed in an inverse ratio; that the one increases at the expense of the other.

This conclusion would appear to be too general, if the observations of Sir Charles Bell on the subject in question are well founded. From a number of experiments and measurements made on the skull of the Negro, as compared with that of the perfect European, he endeavours to prove "that in the Negro the whole of the face is smaller instead of being actually larger, when compared with the brain-case than that of the European;" the jaws, however, he shows, contrasted with other parts of the face are larger. (pp. 35-43.) This leads him to the conclusion that the character of degradation and inferiority in the Negro face is not due to the greater relative development of the whole face, but to the development of those parts of it only which are associated in our minds with bodily and animal appetites. The expansion of this conclusion leads further to the general principle, that as the development of the cranium, the seat of the organ of thought, marks the perfection of the human head, and is associated by us with the possession of intellectual superiority; so also the perfection of the human face consists in the development of those parts of it which are associated with the higher intellectual qualities and superior sentiments. This principle pertains to the face, not only as an instrument of expression, but as an instrument for sound, speech, and articulation.

"In the face" it is justly observed, "there is a character of nobleness observable, depending on the development of certain organs which indicate the prevalence of the higher qualities allied to thought, and therefore human. A great mistake has prevailed in supposing that the expansion of some organs in the face of man, marks a participation in the character of the brute; that the fully developed nose indicates the grovelling propensities, and the extended mouth, the ferocity of the lower animals. Let us correct this misconception by considering the properties or uses of the mouth. It is for feeding certainly, but it is also for speech. Extend the the jaws, project the teeth, widen the mouth, and a carnivorous propensity is declared; but concentrate the mouth, give to the chin fulness and roundness, and due form to the lips; show through them the quality of eloquence, of intelligence, and of human sentiments, and the nobleness is enhanced, which was only in part indicated by the projection of the forehead. Now, look to the antique head and say, is the mouth for masticating, or for speech and expression of sentiment? So of the nose. Here, even Cuvier mistook the principle. The nose on a man's face has nothing in common with the snout of a beast. The prominence of the nose and of the lower part of the forehead, and the development of the cavities in the centre of the face, are all concerned in the *voice.* This is ascertained by the manliness of the voice coming with the full development of these parts. Nothing sensual is indicated by the form of the human nose; although by depressing it, and joining it to the lip,—the condition of the brute,—as in the satyr, the idea of something sensual is conveyed." (p. 31.)

These principles lead to the conclusion that the ancients attained their ideal beauty, and their representation of divinity, by elevating, or slightly exaggerating those features which are strictly human. Of the head of Mercury, for example, an illustration of what may be regarded as simply beautiful, Sir Charles Bell remarks,

"The principle which has been followed in giving beauty to the head of Mercury is obvious here. Whatever is peculiar to the human countenance as distinguishing it from the brute, is enhanced. Not only is the forehead expanded and projecting, and the facial line more perpendicular, but every feature is modelled on the same principle; the ear is small and round; the nostril is eminently human, and unlike that of the beast; the mouth, the teeth, and lips are not such as belong to the brute, nor are they the mere instruments of mastication, but of speech and human expression. So of every part, take them individually, or as a whole; whatever would lead to the resemblance of the brute is omitted or diminished." (p. 59.)

The same general principle is extended to the lower animals, and beauty in them is shown to consist in the adaptation of their organs to their wants and habits. In them the peculiarities of the face and of the whole body depend upon their instincts and propensities, and the more perfect the development of those organs which are subservient to these peculiar habits, the more perfect their beauty of form.

While we agree with the author in the application of this general principle to the lower animals as well as to man, we cannot reconcile it with observation and plain matter of facts, to disinherit them of all but instincts and propensities. We believe with him, that to give human expression to brutes, as Rubens for example, has done, in his painting of Daniel in the Lion's den, is not less absurd than repulsive to good taste; but while we deny them human emotions in the full extent, we cannot for a moment admit that they are destitute of mental qualities. It was, doubtless, a fanciful analogy with the expanded forehead of man, that led the ancients to dignify the owl as the companion of Minerva,—the bird of wisdom;—but it requires no exercise of fancy, but only a habit of observation and of intercourse, to perceive in the countenances of our humble and faithful companions, the perfect expression of affection and of reason. Their acts are in many instances explicable on no other supposition than that they are influenced and directed by both qualities, and surely their faces rightly understood will indicate their influence as well as that of the natural propensities by which they are peculiarly characterized.

The principles which we have briefly sketched form the basis of the first two essays, through which, we may say, they are scattered. They are illustrated, as indeed, the whole work is, to a greater or less extent, by pleasing and interesting remarks on the celebrated works of the great masters which the author had examined. We cannot but complain of a want of systematic arrangement, and a degree of amplication and recapitulation extending not only to those essays, but throughout the work.

The second essay contains, at its commencement, also a sketch of the changes which the form of the skull undergoes from infancy to old age; and closes with a survey of the national peculiarities in the form of the head to be met with among the different varieties of mankind. With regard to the form of the head of the ancient Romans, the observations of the author are interesting, and coincide entirely with those of Bishop Wiseman. He concludes that the forms delineated in the majority of their statues and bas reliefs, were formed from Grecian models, and give no idea of the true characters of the Roman head. These are to be distinguished in the imperial busts, and reclining statues of the sarcophagi, or, at the present time, in the Burgesses or among the Galleortti, and lead to the conclusion that the characters of the true Roman, contrary to common belief, were "a large flat head, a low and wide forehead, a face broad and square, a short thick neck, and a stout and broad trunk." (p. 76.)

The third essay is devoted to an examination of those sources of expression in the countenance which are not directly expressive of the acts of the mind, but involuntary and sympathetic.

"We can readily conceive," says Sir Charles, "why a man stands with eyes intently fixed on the object of his fears, the eyebrows elevated to the utmost, and the eye largely uncovered; or why, with hesitating and bewildered steps, his eyes are rapidly and wildly in search of something. In this we only perceive the intense application of his mind to the object of his apprehensions—its direct influence on the outward organ. But observe him further: there is a spasm on his breast, he cannot breathe freely, the chest is elevated, the muscles of his neck and shoulders are in action, his breathing is short and rapid, there is a gasping and a convulsive motion of his lips, a tremor on his hollow cheek, a gulping and catching of his throat; and why does his heart knock at his ribs, while yet there is no force of circulation? for his lips and cheeks are ashy pale." (p. 88.)

The explanation of those symptoms of emotion, Sir Charles Bell finds in the sympathy between the mind and the heart. The derangement of the heart's action, produced by the influence of passion, disturbs the circulation through the lungs, the breathing becomes affected, the whole apparatus connected with the respiration and the voice is influenced, and thus the muscles of respiration become the organs of expression. In this manner are explained the trembling lips, the dilated nostrils, the loss of voice, and the fixed or expanded chest which accompany strong emotion.

The beautiful and ingenious theory, commonly called "the respiratory system of Sir Charles Bell," in our opinion falls as far short of the truth as an exposition of the involuntary indications of passion, as we believe it goes beyond the truth as an exposition of part of the anatomy of the nervous system. The muscles of respiration are undoubtedly organs of expression, and they are so, probably, through the medium of the heart, in the manner Sir C. Bell has so well explained and illustrated. But these are only examples of a sympathy which affects the whole frame and makes every organ an instrument of expression. The same emotions which produce the palpitations of fear, and the speechless agony of terror, which make us sob with anguish, or laugh with joy, or breathless with delight, produce also the powerless grasp and trembling knees, the tears of woe, and of excess of happiness. Fear relaxes every limb; nor does it stop with the muscles of animal life; it affects those also of involuntary motion. Fear and anger blanch, while shame crimsons the face. One emotion makes the tears fall, another makes the mouth parched, and the voice inarticulate not so much from paralysis of its muscles, as from want of moisture. In short, no organ of the body is beyond the influence of the passions. Of this innumerable illustrations might be adduced. The increased secretion from the skin, or mucous membranes, under the influence of certain emotions; the sudden development of jaundice from fear; the whitening of the hair from grief or anxiety, the influence of the mind in the production or cure of disease, all evince the extent to which the whole frame is affected by the mental emotions. To regard the muscles of respiration as, *par excellence*, the organs of expression, is to take a limited view of the subject. They are affected more readily, and to a greater extent than other muscles, and this, perhaps, because they are exposed to a twofold influence, the direct influence of the passion, and the indirect influence produced by the derangement of the heart's action, and that of the circulation through the lungs.

In connexion with this subject, and as affording an additional illustration of the justice of our criticism, we may refer to the movements of the eye under the influence of emotion. Granting that the fourth pair (*pathetici*) could be traced to the so-called respiratory tract of the nervous system, how does that fact explain the upward glancing of the eyes in certain emotions, as stated by Sir Charles Bell? To resort, as he has done, to the supposition that in this instance the impression conveyed through the fourth nerve to the superior oblique muscles produces *relaxation* of that muscle, even if it could explain the phenomenon, is only another illustration of the extent to which a favorite hobby can be driven, for that movement is produced not by the superior but by the inferior oblique muscle of the eyeball. The inferior oblique is supplied by the common motor nerve of the eyeball: how does this fact consist with the theory that it is the special function of the respiratory muscles and those associated with them, to afford a language to passion.

The Fourth and Fifth Essays are devoted to a description of the muscles of the face in man and the lower animals, in relation to expression. The anatomical descriptions, we think, are somewhat meagre, and the woodcuts by which they are illustrated rather poor. The description of their uses, and their relations to speech and expression, is ingenious and interesting, and is illustrated by references to familiar facts, and to well-known works of art.

Certain muscles in the human face are shown to be exclusively designed for being instrumental in expression. These are mostly the muscles of the eyebrow and forehead: "Frons hominis tristitiæ, hilaritatis, clementiæ, servitatis, index est." (Pliny.) Some muscles are partly instrumental in breathing, but mostly in speech; these also are peculiarly characteristic of man, and main organs of expression. So far as these muscles are connected with respiration, they are common also to the lower animals, and give a peculiar character to certain animals distinguished by their fleetness and activity, as is seen, for example, in the dilated nostrils of the horse. The muscles of the cheeks and lips in the lower animals have special relations to their habits: in the gramnivorous animals they are adapted for covering and uncovering the incisors in gathering food, in the carnivora for retracting the angles of the mouth and uncovering the canine teeth while the animal prepares to seize its prey. The development of those muscles respectively gives to each class of animals their peculiar characters.

Man, being omnivorous, partakes, to a certain degree, of the characters of both these classes of animals in the development of the muscles of the mouth; but in him the mouth is also an instrument of speech, and it is from its adaptation for this function that it derives those characters which are peculiarly human. The human countenance is thus capable of expressing the rage of the most ferocious animals, and the gentleness of the most timid; while at the same time, by a *special* provision for speech, and the expression of mental emotion, it becomes an index of the infinite variety of thoughts and feelings of which the mind is susceptible.

From the illustration of these principles Sir Charles Bell passes, in the subsequent essays, to the description of the outward manifestations of the various passions. His own graphic delineations of passion, and his illustrative sketches are accompanied by references to the celebrated works of the great masters, and citations from the animated and accurate representations of Virgil, Tasso, Ariosto, Spencer, Shakspeare, and Milton. An explanation of the action of the various muscles in producing the expression of those passions, is at the same time given; and some general rules for their representation by the artist are thus elicited.

The utility of such rules and descriptions is, we believe, apt to be very much overrated. To be told that certain muscles which are described elevate, and certain others depress the lips, and that the angles of the mouth are drawn upwards in laughter, downwards in grief, and outwards in rage, may direct the artist to a certain extent. But there is much in the expression of the various passions and their modifications which description can never convey, and which no rules can comprise. A natural talent for imitation, and a habit of careful observation, with a power of realizing in his own mind a vivid conception of the emotion to be portrayed, can alone enable the artist to arrive at eminence in the delineation of the passions. The study of anatomy, and the descriptions of the poets may aid in the study of nature, and quicken the powers of observation; but in relation to the higher departments of art it may be said of the artist as of the poet, "*nascitur, non fit.*" To the artist we believe that even the study and observation of nature in reference to this subject, is of use simply by enabling him to read accurately in the countenance the emotions of the mind. By the aid of certain rules we may at once delineate the general features of any class of emotions; but the details which give spirit and character to the whole, or which indicate the mixed emotions or modifications of feeling which he wishes to depict, are worked out, not by rule or by the recollection of certain lines, but by a method of trial and error, as mathematicians would call it, by which he gradually develops the expression suited to his subject. We cannot look at the admirable sketch at p. 178 of the work before us without a conviction that the description was made for the sketch, and not the sketch for the description. The rules and principles have yet to be discovered by which an artist may be enabled to delineate the workings of the human mind. Notwithstanding all that has been written on the subject, we believe that he must yet rely chiefly on the vivid conceptions of his own mind to guide him intuitively to the delineation of passion.

We have dwelt thus long on this work, not only because it comprises several interesting inquiries of a philosophical and physiological character, and because it was the favorite occupation of a distinguished physiologist, but because we believe the subject is one of interest, and of some practical importance to the physician as well as to the artist. Good taste and sound judgment in relation to the fine arts form part of the necessary accomplishments of a gentleman. They are certainly not less necessary if he is also a physician. The direct and obvious advantages of being able to delineate with the pencil the form and appearances of diseased structures, or the daily objects of scientific observation, require no comment. But the study of the arts of design, pursued even to the highest department—the delineation of human passion —has its advantages; it sharpens the powers of observation, and quickens the eye for the detection of those delicate changes in form, colour, or expression, by which the diagnosis of the accomplished physician is so promptly and correctly made.

In conclusion, we would say that this is a beautiful book, in every sense of the word; and, like everything beautiful, delightful. It is not, to be sure, what in vulgar language—language unworthy of science—is called a "practical book;" but it is one which no physician or surgeon, with any pretensions to literary taste, can pass by unread; and assuredly it is one which no medical library or medical book-club in the United Kingdom can do without.

